# Multi‐omics analysis identifies a major histocompatibility complex class II‐associated antigen‐presenting cancer‐associated fibroblast‐like state linked to the nuclear factor erythroid 2‐related factor 2‐karyopherin subunit beta 1 axis in nonsmall cell lung cancer

**DOI:** 10.1002/ccs3.70074

**Published:** 2026-06-04

**Authors:** Fei Zheng, Ruoying Deng, Ran Hou, Lei Hong, Yanzhi Cui, Yibing Liu

**Affiliations:** ^1^ Department of Oncology The Fourth Hospital of Hebei Medical University Shijiazhuang China

**Keywords:** antigen‐presenting CAF‐like state, cancer‐associated fibroblasts, non‐small cell lung cancer, NRF2‐KPNB1 axis, PD‐1 blockade

## Abstract

To define the transcriptional features, spatial distribution, and immune‐related properties of an antigen‐presenting cancer‐associated fibroblast (CAF)‐like state in nonsmall cell lung cancer (NSCLC), and to evaluate its relationship with the nuclear factor erythroid 2‐related factor 2 (NRF2)‐karyopherin subunit beta 1 (KPNB1) axis, multi‐omics analyses integrating single‐cell RNA sequencing, spatial transcriptomics, and bulk transcriptomic data were performed. Functional validation used primary CAFs from patients with NSCLC, including NRF2 perturbation, KPNB1 knockdown, T‐cell co‐culture assays, and an orthotopic murine lung cancer model treated with antiprogrammed cell death protein 1 (anti‐PD‐1)‐based combinations. A major histocompatibility complex class II (MHC‐II)‐associated antigen‐presenting CAF‐like (apCAF‐like) state was identified in NSCLC. It showed increased antigen‐presentation‐related molecules, limited co‐stimulatory molecule expression, and inferred communication involving MHC‐II, transforming growth factor‐β, and C‐X‐C motif chemokine ligand pathways. Higher KPNB1 expression was associated with poorer survival, lower immune infiltration, and lower ImmuneScore. In primary CAFs, NRF2 activation increased KPNB1 and reduced class II MHC transactivator, major histocompatibility complex class II DR alpha (HLA‐DRA), and MHC‐II‐related signals, whereas NRF2 inhibition showed the opposite pattern. KPNB1‐low CAFs enhanced T‐cell activation and cytokine release, and KPNB1 knockdown enriched antigen‐presentation‐related transcriptional programs. In vivo, inhibition of the NRF2‐KPNB1 axis enhanced response to anti‐PD‐1. NSCLC contains an MHC‐II‐associated apCAF‐like state linked to the NRF2‐KPNB1 axis and altered response to PD‐1 blockade.

## Graphical abstract

NSCLC contains an MHC‐II‐associated antigen‐presenting CAF‐like (apCAF‐like) state regulated by the NRF2‐KPNB1 axis. NRF2/KPNB1 signaling suppresses CIITA‐dependent antigen presentation and T‐cell activation, whereas inhibition of this pathway enhances antitumor immunity and improves responsiveness to anti‐PD‐1 therapy.

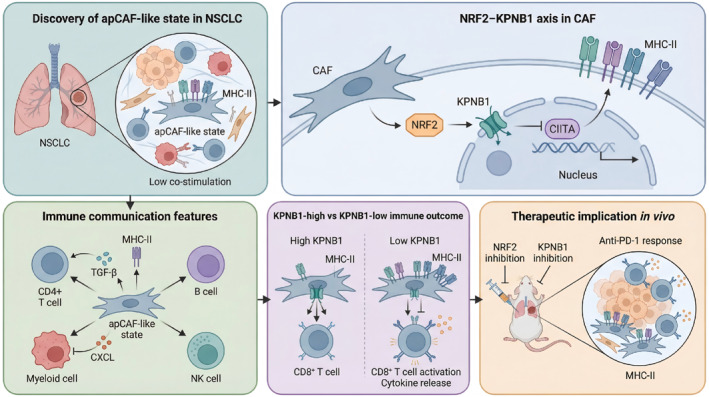

## 1 INTRODUCTION

Nonsmall cell lung cancer (NSCLC) remains one of the leading causes of cancer incidence and mortality worldwide, posing a major threat to human health.^1^, ^2^ Immune checkpoint inhibitors have improved clinical outcomes in a subset of patients with NSCLC.^3^, ^4^ However, the overall response rate remains below 30%, and both primary and acquired resistance, together with immune exclusion, are common.^5^, ^6^ These observations indicate that determinants of immunotherapy response extend beyond tumor cells themselves, and that nonmalignant components of the tumor microenvironment (TME) are also likely to play important roles.^7^, ^8^

Cancer‐associated fibroblasts (CAFs) are major stromal components of the TME and have been implicated in extracellular matrix remodeling, inflammatory regulation, and immune suppression.^9^, ^10^ Previous studies have largely emphasized the ability of CAFs to restrict immune‐cell infiltration and activation through secretion of immunosuppressive mediators, such as transforming growth factor‐β (TGF‐β) and interleukin‐6 (IL‐6), or through formation of stromal barriers.^11^, ^12^ However, with the development of single‐cell approaches CAFs are no longer viewed as a uniform population, but rather as a collection of transcriptionally heterogeneous and plastic cell states that may carry distinct immune‐related functions.^13^, ^14^, ^15^, ^16^

Recent studies have described a CAF state characterized by expression of major histocompatibility complex class II (MHC‐II)‐related molecules, commonly referred to as antigen‐presenting CAFs (apCAFs).^17^, ^18^ Within pan‐cancer CAF classification frameworks, this state has been recognized as a relatively distinct transcriptional subtype,^16^ extending current views of CAF‐mediated immune regulation.^19^ However, its prevalence in NSCLC, transcriptional definition, spatial distribution and immunological consequences remain incompletely resolved.^20^, ^21^ Importantly, expression of MHC‐II molecules does not necessarily imply functional antigen presentation; in the absence of co‐stimulatory signals, such a state may instead reflect an incomplete or tolerogenic immune‐related phenotype.

Nuclear factor erythroid 2‐related factor 2 (NRF2) is a central transcriptional regulator of oxidative stress responses and has been linked to metabolic adaptation, therapy resistance and microenvironmental remodeling in multiple tumor types.^22^, ^23^ Emerging evidence suggests that NRF2 activity is not confined to tumor cells and may also influence stromal‐cell states, including CAF phenotypes and outputs.^24^, ^25^ However, whether NRF2 is associated with MHC‐II‐related CAF states in NSCLC remains unclear.^26^, ^27^, ^28^, ^29^

Karyopherin subunit beta 1 (KPNB1) is a key nuclear transport protein that mediates the nucleocytoplasmic trafficking of multiple transcriptional regulators, including members of the signal transducer and activator of transcription family, nuclear factor‐κB, and class II MHC transactivator (CIITA), thereby influencing cellular stress and immune‐response programs.^30^, ^31^ In several solid tumors, KPNB1 has been associated with tumor progression and immune evasion,^32^, ^33^ but its role in CAFs remains poorly defined.^34^, ^35^ Given that expression of MHC‐II‐related molecules depends on nuclear transcriptional networks involving CIITA, KPNB1 may be linked to the establishment or maintenance of MHC‐II‐associated CAF states.^14^, ^36^ Previous studies have also suggested that KPNB1 may be regulated by NRF2, raising the possibility that an NRF2‐KPNB1 axis contributes to immune‐related CAF phenotypes.^37^, ^38^, ^39^

Multi‐omics approaches, including single‐cell transcriptomics, spatial transcriptomics, single‐cell assay for transposase‐accessible chromatin using sequencing (scATAC‐seq) and proteomics, provide a framework for resolving the distribution, states, and functions of CAF subpopulations.^40^, ^41^ Combined with spatial proximity analyses and functional experiments, these approaches enable assessment of the relationship between CAF states, local immune niches and treatment response.^9^, ^14^, ^42^, ^43^ On this basis, the present study integrates single‐cell transcriptomics, spatial transcriptomics, The Cancer Genome Atlas (TCGA) cohort, primary CAF functional assays, and mouse models to evaluate the presence of an MHC‐II‐associated apCAF‐like state in NSCLC, define its transcriptional and spatial features, and examine the relationship of the NRF2‐KPNB1 axis to this state and to antiprogrammed cell death protein 1 (anti‐PD‐1) treatment response.

## 2 MATERIALS AND METHODS

### 2.1 Acquisition and preprocessing of single‐cell transcriptomic data

The publicly available single‐cell RNA‐sequencing (scRNA‐seq) dataset of NSCLC, GSE131907, containing cells derived from tumor and adjacent normal tissues, was downloaded from the Gene Expression Omnibus database. Analyses were performed using the raw count matrices. Data were processed with Seurat (v4.0.5) for quality control and downstream analyses. Cells with fewer than 200 detected genes, more than 6000 detected genes, or mitochondrial transcript content >10% were excluded. A total of 41,863 high‐quality cells were retained for subsequent analyses.

### 2.2 Normalization, dimensionality reduction and clustering

Expression matrices were normalized using NormalizeData(), and highly variable genes were identified using FindVariableFeatures(). Principal component analysis was used for linear dimensionality reduction, followed by uniform manifold approximation and projection (UMAP) based on the top 30 principal components. Cell clustering was performed using FindNeighbors() and FindClusters() at a resolution of 0.6. Cell‐type annotation was guided by canonical marker genes reported previously, including CD3D, CD79A and PECAM1, and refined using differentially expressed genes (DEGs) identified in the present study.

### 2.3 Fibroblast subpopulation analysis

Fibroblasts were extracted from the global cell atlas and subjected to secondary unsupervised clustering using FindClusters() at a resolution of 0.4. UMAP was used to visualize fibroblast subclusters. Differential expression analysis was conducted using FindMarkers() with the Wilcoxon rank–sum test, applying thresholds of log_2_ fold change >0.25 and adjusted *p* < 0.05. Fibroblast states were annotated according to differential expression patterns and established marker genes, yielding subpopulations, including apCAF‐like cells, inflammatory CAFs (iCAFs), myofibroblastic CAFs (myCAFs), and normal fibroblasts (Normal_FB).

### 2.4 Module scoring, enrichment analysis and gene‐expression comparison

Module scores for apCAF‐like signatures and antigen processing and presentation programs were calculated using Seurat‐based module scoring. DEGs from fibroblast subclusters were subjected to gene ontology (GO) enrichment analysis using clusterProfiler (v4.0), with particular focus on immune‐related terms including antigen processing and presentation and MHC class II protein complex assembly. Expression patterns of NFE2L2, KPNB1, major histocompatibility complex class II DR alpha (HLA‐DRA), CD74, and related genes were visualized using UMAP, feature plots, and violin plots. For selected analyses, fibroblasts were stratified according to KPNB1 expression, and the expression of antigen‐presentation‐related genes, including HLA‐DRA, CD74 and CIITA, was compared between groups.

### 2.5 Pseudotime trajectory analysis

Fibroblast populations were analyzed by pseudotime trajectory inference using Monocle 2. High‐quality fibroblast cells and Seurat‐normalized expression matrices were used as input. Genes selected for trajectory ordering were obtained from differential expression analysis, using thresholds of log_2_ fold change >0.25 and adjusted *p* < 0.05. Dimensionality reduction was performed using DDRTree, and cells were ordered along pseudotime. Expression dynamics of representative genes, including ACTA2, CD74, HLA‐DRA, IRF1, KPNB1 and NFE2L2, were visualized across the inferred trajectory.

### 2.6 Survival and immune‐score analysis in TCGA

RNA‐seq expression matrices and matched clinical annotations for lung adenocarcinoma (LUAD; *n* = 522) and lung squamous cell carcinoma (LUSC; *n* = 504) were obtained from TCGA, yielding 1026 evaluable samples. Expression values were transformed as log_2_(FPKM + 1). Samples were stratified into high‐ and low‐KPNB1 groups according to the median KPNB1 expression level. Overall survival was analyzed using Kaplan–Meier curves and the log–rank test. Cox proportional hazards analysis was performed using clinicopathological covariates including pathological T stage and N stage.

Bulk immune features were further assessed using single‐sample gene set enrichment analysis (ssGSEA) and the ESTIMATE algorithm. Immune infiltration scores for immune‐cell subsets and ImmuneScore values were correlated with KPNB1 expression using Pearson's or Spearman's correlation, as appropriate.

### 2.7 Ligand–receptor interaction analysis

Cell–cell communication analysis was performed using CellChat (v1.1.3) on the GSE131907 scRNA‐seq dataset. The apCAF‐like population and major immune‐cell populations, including T lymphocytes, B lymphocytes, myeloid cells, and natural killer (NK) cells, were extracted according to Seurat annotations and incorporated into a CellChat object. Potential ligand–receptor pairs were inferred using the Secreted Signaling database. Communication probabilities were calculated using computeCommunProb() and filtered using filterCommunication() with a significance threshold of *p* < 0.05.

Network centrality analysis was performed using netAnalysis_signalingRole() to quantify sender, receiver, mediator, and influencer roles. Downstream analyses focused on the MHC‐II, TGF‐β and C‐X‐C motif chemokine ligand (CXCL) signaling pathways, which were visualized using centrality heatmaps, chord diagrams and ligand–receptor bubble plots.

### 2.8 Pathway enrichment analysis of the apCAF‐like population

Pathway‐enrichment analyses were performed to characterize transcriptional programs of the apCAF‐like population relative to other CAF subtypes. Gene set enrichment analysis (GSEA) was conducted using clusterProfiler (v4.8.0) with Hallmark, Kyoto encyclopedia of genes and genomes (KEGG), and Reactome gene sets. Enrichment plots were generated for pathways, including HALLMARK_INTERFERON_GAMMA_RESPONSE, KEGG_ANTIGEN_PROCESSING_AND_PRESENTATION, HALLMARK_ALLOGRAFT_REJECTION, HALLMARK_INFLAMMATORY_RESPONSE, and [Reactome] MHC Class II Antigen Presentation. Ridgeplots were used to summarize enriched Hallmark pathways.

### 2.9 Spatial transcriptomics and spatial association analysis

Spatial transcriptomic data from NSCLC (E‐MTAB‐13530) were analyzed using Seurat and SPATA2 for spatial clustering, tissue mapping, and regional visualization. Unsupervised clustering identified spatial subclusters across the tissue section. Bivariate Moran's I analysis was used to assess the spatial correlation between CD8A and ITGAX. Ripley's K analysis, using the L(*r*) − *r* transformation, was used to evaluate the spatial distribution of KPNB1‐high spots. Spatial overlays were generated to assess the regional relationship between CXCL12‐high and KPNB1‐high spots. Dot plots of representative genes across spatial clusters and spatial mapping of KPNB1‐associated, NRF2‐related and antigen‐presentation‐related signals were used for regional comparison.

### 2.10 Primary human CAFs

Primary CAFs were isolated from fresh surgical specimens from patients with NSCLC (*n* = 150). Tissue samples were obtained from patients with primary LUAD or squamous cell carcinoma, with a male‐to‐female ratio of approximately 1.2:1 and a median age of 62 years. Cells were isolated and expanded in‐house within 72 h of surgery. CAF identity was confirmed by immunophenotyping, with positivity for fibroblast activation protein (FAP), podoplanin (PDPN), α‐smooth muscle actin, and negativity for epithelial cell adhesion molecule and CD45. Cells were used at low passage (passage 3 or below). Mycoplasma contamination was excluded using the MycoAlert Mycoplasma Detection Kit (Lonza, LT07‐318).

### 2.11 Murine Lewis lung carcinoma cell line

The murine Lewis lung carcinoma cell line LL/2 (Research Resource Identifier, RRID: CVCL_4358) was obtained from the American Type Culture Collection (ATCC) in June 2024. Cells were cultured in Dulbecco's modified Eagle medium (DMEM) supplemented with 10% fetal bovine serum and 1% penicillin–streptomycin. Mycoplasma testing was performed regularly, and the cell line was authenticated upon receipt.

### 2.12 Primary CAF culture, gene perturbation and immune co‐culture

Fresh NSCLC tissues were digested with collagenase I at 1 mg/mL and deoxyribonuclease I (DNase I) at 100 μg/mL at 37°C for 1.5 h, filtered through a 70 μm mesh, and enriched for CAFs using immunomagnetic beads against FAP and PDPN. Cells were expanded short term in DMEM/F12 medium. For stratification experiments, CAFs were sorted into KPNB1‐low and KPNB1‐high populations using a KPNB1‐Alexa647 antibody by fluorescence‐activated cell sorting.

For NRF2 perturbation, CAFs were transfected with NRF2‐targeting small interfering RNA (siRNA) or subjected to CRISPR activation (CRISPRa)‐mediated NRF2 activation. RNA and protein were collected 48 h after perturbation, and expression of KPNB1, CIITA, HLA‐DRA and CD74 was assessed by quantitative polymerase chain reaction and western blotting.

For immune co‐culture assays, CD8^+^ T cells were isolated from peripheral blood using CD8 magnetic beads, with purity >95%, and co‐cultured with CAF subgroups at an effector‐to‐target ratio of 1:2 for 48 h. T‐cell activation was analyzed by flow cytometry using CD3, CD8, CD69 and CD25. Cytokines in supernatants, including interferon‐γ (IFN‐γ) and tumor necrosis factor‐α, were quantified by enzyme‐linked immunosorbent assay. Contact dependence was further assessed using Transwell assays.

### 2.13 KPNB1 knockdown and bulk RNA‐seq

Primary CAFs were transfected with KPNB1‐targeting siRNA (siKPNB1#1/#2; GenePharma; final concentration 50 nM) using Lipofectamine RNAiMAX. Total RNA was extracted after 48 h using TRIzol. RNA integrity was confirmed, with RNA integrity number values of at least 7.0, and libraries were prepared using the NEBNext Ultra II RNA Library Prep Kit with poly(A) selection. Paired‐end sequencing with a read length of 150 base pairs was performed on the Illumina NovaSeq 6000 platform, generating approximately 50 million clean reads per sample. Reads were aligned to the human reference genome hg38 using STAR (v2.7), and gene counts were generated using featureCounts. Differential expression analysis was performed using DESeq2 with thresholds of false discovery rate (FDR) <0.05 and absolute log_2_ fold change >1. GO, KEGG and GSEA analyses were then performed to characterize transcriptional changes associated with KPNB1 knockdown.

### 2.14 Orthotopic NSCLC mouse model and treatment

C57BL/6J mice aged 6–8 weeks (*n* = 75; equal numbers of males and females) were housed under specific‐pathogen‐free conditions. All procedures were approved by the Institutional Animal Ethics Committee. Mice were randomly assigned to five groups (*n* = 15 per group): control, anti‐PD‐1, anti‐PD‐1 + CDDO‐Me (30 mg/kg), anti‐PD‐1 + siNRF2, and anti‐PD‐1 + importazole (20 mg/kg).

Orthotopic lung tumors were established by injection of 2 × 10^6^ LL/2 cells into the left upper lung lobe. Antiprogrammed cell death protein 1 (anti‐PD‐1) antibody was administered at 10 mg/kg by intraperitoneal injection twice weekly. CDDO‐Me and Importazole were administered intraperitoneally for 3 weeks, and siNRF2 was administered according to the intervention schedule used for in vivo NRF2 silencing. Tumor growth was monitored weekly. Body weight, clinical status, and survival were recorded throughout the experiment.

At day 21, tumors were collected for histological and molecular analyses. Paraffin‐embedded sections were used for immunohistochemistry (IHC), and additional tissue was processed for isolation of tumor‐infiltrating lymphocytes and CAF‐enriched fractions. RNA and protein from tumor‐derived CAFs were used to assess NRF2–KPNB1 signaling and MHC‐II‐related molecules.

### 2.15 Statistical analysis

All experiments were performed with at least three biological replicates unless otherwise indicated. Data are presented as mean ± standard deviation (SD) or median ± interquartile range, as appropriate. Statistical analyses were conducted using GraphPad Prism (v9.0) and R (v4.2.2). Two‐group comparisons were performed using a two‐tailed Student's *t*‐test or Mann–Whitney *U* test, depending on data distribution. Multiple‐group comparisons were analyzed using one‐way analysis of variance with post hoc multiple‐comparison correction. Kaplan–Meier survival curves were compared using the log–rank test. Pearson's or Spearman's correlation coefficients were used according to the data structure. Spatial association analyses were performed using Moran's I and Ripley's K. All tests were two‐sided, and *p* < 0.05 was considered statistically significant.

## 3 RESULTS

### 3.1 Transcriptional identification of an MHC‐II‐associated fibroblast state and its inferred immune interaction features in NSCLC

**FIGURE 1:**
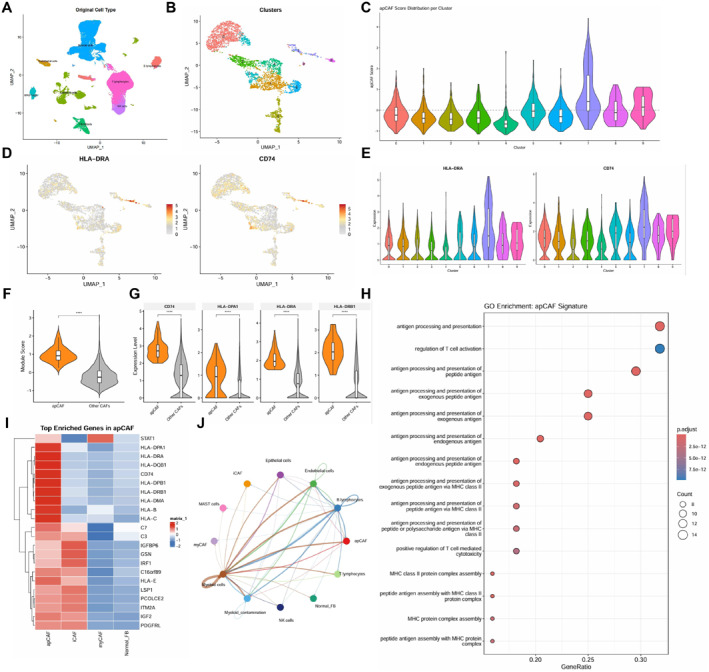
Transcriptional identification of apCAF‐like fibroblasts in NSCLC. (A) UMAP plot of the major cell types in GSE131907. (B) UMAP plot of reclustered fibroblasts showing 10 subclusters (Cluster 0–9). (C) apCAF signature scores across fibroblast subclusters. (D) Feature plots of HLA‐DRA and CD74 in reclustered fibroblasts. (E) Expression of HLA‐DRA and CD74 across fibroblast subclusters. (F) Comparison of antigen processing and presentation module scores between apCAF‐like cells and other CAFs; Wilcoxon test, ****p < 0.0001. (G) Expression of CD74, HLA‐DPA1, HLA‐DRA and HLA‐DRB1 in apCAF‐like cells and other CAFs; Wilcoxon test, ****p < 0.0001. (H) GO enrichment analysis of genes upregulated in apCAF‐like cells. (I) Heatmap of representative genes across fibroblast states. (J) CellChat‐inferred intercellular communication network for the MHC‐II signaling pathway. apCAF, antigen‐presenting cancer‐associated fibroblast; GO, gene ontology; MHC‐II, major histocompatibility complex class II; NSCLC, nonsmall cell lung cancer; UMAP, uniform manifold approximation and projection.

Single‐cell transcriptomic analysis of GSE131907 resolved the major cellular compartments in NSCLC tumor and adjacent normal tissues (Figure 1A). Re‐clustering of the fibroblast compartment identified 10 transcriptionally distinct subclusters (Cluster 0–9) (Figure 1B).

Module scoring based on an apCAF gene signature showed that Cluster 7 had a relatively high apCAF signature score (Figure 1C). Feature plots of HLA‐DRA and CD74 showed that MHC‐II‐related transcripts were primarily localized to Cluster 7 (Figure 1D). Violin plots further showed that HLA‐DRA and CD74 expression were higher in Cluster 7 than in most other fibroblast subclusters (Figure 1E). On this basis, Cluster 7 was defined as an apCAF‐like population.

Compared with other CAFs, the apCAF‐like population showed a higher antigen processing and presentation module score (*p* < 0.0001) (Figure 1F). CD74, HLA‐DPA1, HLA‐DRA, and HLA‐DRB1 were also increased in the apCAF‐like population (all *p* < 0.0001) (Figure 1G). GO enrichment analysis showed that the upregulated genes were mainly associated with antigen processing and presentation, regulation of T cell activation, MHC class II protein complex assembly, and related processes (Figure 1H). The heatmap further showed enrichment of HLA‐DRA, CD74, HLA‐DPA1, HLA‐DRB1, and IRF1 in the apCAF‐like population, whereas iCAFs were enriched for C3 and C7 (Figure 1I).

CellChat analysis showed that, within the MHC‐II signaling network, the apCAF‐like population displayed inferred communication edges directed toward T lymphocytes, whereas iCAFs, myCAFs, and Normal_FB did not show the same pattern (Figure 1J). These data define an apCAF‐like fibroblast state with MHC‐II‐associated transcriptional features in NSCLC and suggest its potential involvement in T cell‐related signaling interactions.

### 3.2 Pseudotime distribution of CAF states and associated gene expression dynamics

**FIGURE 2:**
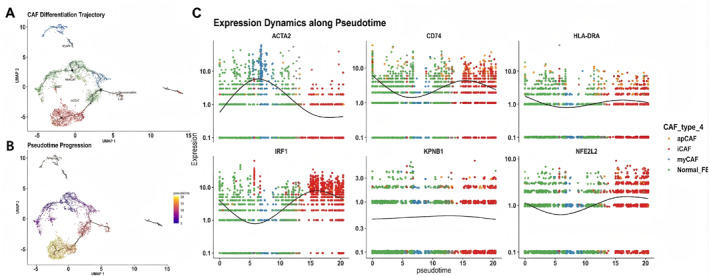
Pseudotime trajectory of CAF subtypes and associated gene expression dynamics. (A) Monocle 2 pseudotime trajectory colored by CAF subtype, including Normal_FB, myCAF, iCAF and the apCAF‐like population. (B) The same trajectory colored by pseudotime value, with dark purple indicating early states and yellow indicating late states. (C) Gene‐expression dynamics along pseudotime for ACTA2, CD74, HLA‐DRA, IRF1, KPNB1 and NFE2L2, shown as scatter distributions with fitted curves; the *x* axis indicates pseudotime and the *y* axis indicates expression level. apCAF, antigen‐presenting cancer‐associated fibroblast; iCAFs, inflammatory CAFs; KPNB1, karyopherin subunit beta 1; myCAFs, myofibroblastic CAFs; Normal_FB, normal fibroblasts.

Monocle 2 pseudotime analysis resolved a continuous trajectory of CAF state transitions (Figure 2A). When colored by cell‐state annotation, Normal_FB, myCAFs, iCAFs, and the apCAF‐like population showed distinct distributions along the branched structure, with the apCAF‐like population located predominantly at the terminus of one branch, whereas myCAFs and iCAFs occupied different trajectory segments (Figure 2A).

When colored by pseudotime value, early and late cellular states were arranged continuously along the same trajectory, with higher pseudotime values concentrated in the terminal region containing the apCAF‐like population (Figure 2B). This distribution positioned the apCAF‐like population within the late segment of the CAF state‐transition trajectory.

Fitted gene‐expression trends along pseudotime showed that ACTA2 reached higher levels early and then declined, whereas CD74 increased again in the intermediate‐to‐late phase; HLA‐DRA showed limited variation across the trajectory (Figure 2C). Among the regulatory factors, IRF1 increased more prominently in the late segment, whereas KPNB1 varied little across the trajectory; NFE2L2 showed a modest upward trend toward the later phase (Figure 2C). These data define the pseudotime position of the apCAF‐like population during CAF state transitions and show distinct dynamic patterns for MHC‐II‐related genes and selected regulatory factors along the trajectory.

### 3.3 Association of KPNB1 expression with survival outcome in NSCLC

**FIGURE 3:**
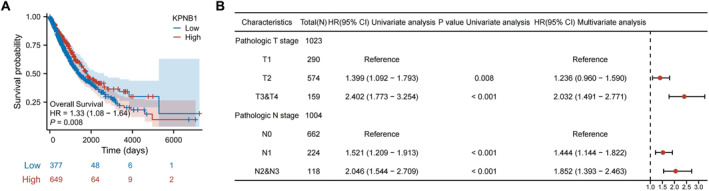
Association of KPNB1 expression with survival outcome in NSCLC. (A) Kaplan–Meier overall survival curves stratified by high and low KPNB1 expression; blue indicates the low‐expression group, red indicates the high‐expression group; shaded areas indicate confidence intervals. (B) Forest plot of Cox proportional hazards analysis showing the associations of pathological T stage and N stage with survival risk; points indicate hazard ratios and horizontal lines indicate 95% confidence intervals. KPNB1, karyopherin subunit beta 1; NSCLC, nonsmall cell lung cancer.

Kaplan–Meier analysis in the TCGA LUAD/LUSC cohort showed that the high‐KPNB1 group had a lower overall survival probability than the low‐expression group (*p* = 0.008) (Figure 3A). This result associates higher KPNB1 expression with poorer survival outcome.

Cox proportional hazards analysis incorporated pathological T stage and N stage as clinicopathological variables (Figure 3B). In this model, higher T stage and nodal stage were associated with increased risk, indicating that tumor burden and pathological progression remained major determinants of survival (Figure 3B).

Single‐cell expression patterns are shown in Figure S1. Relative to normal tissue, KPNB1 expression showed an overall increase across multiple major cell types in tumor samples, including epithelial cells, fibroblasts, myeloid cells, and NK cells (Figure S1A). Across major cell types in the TME, KPNB1 was detectable in epithelial cells, fibroblasts, myeloid cells, and NK cells, whereas expression was generally lower in lymphocyte populations (Figure S1B). These data indicate that KPNB1 expression is not restricted to a single lineage but is distributed across tumor cells and multiple stromal cell populations.

### 3.4 MHC‐II‐associated communication features and limited co‐stimulatory molecule expression in the apCAF‐like population

**FIGURE 4:**
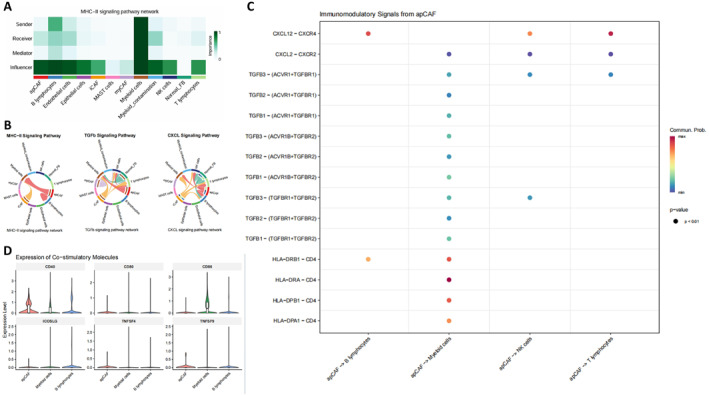
Inferred communication features between the apCAF‐like population and immune cells. (A) Heatmap of network centrality in the MHC‐II signaling pathway, showing the relative scores of different cell populations in the sender, receiver, mediator, and influencer dimensions. (B) Chord diagrams showing inferred intercellular communication networks in the MHC‐II, TGF‐β and CXCL signaling pathways; connecting lines indicate inferred communication relationships between cell populations. (C) Bubble plot of ligand–receptor interactions derived from the apCAF‐like population; dot color indicates inferred communication probability and dot size indicates *p* value. (D) Expression distributions of the co‐stimulatory molecules CD40, CD80, CD86, ICOSLG, TNFSF4, and TNFSF9 in the apCAF‐like population, myeloid cells and B lymphocytes. apCAF, antigen‐presenting cancer‐associated fibroblast; CXCL, C‐X‐C motif chemokine ligand; MHC‐II, major histocompatibility complex class II; TGF‐β, transforming growth factor‐β.

CellChat analysis was used to infer potential communication networks between the apCAF‐like population and immune cells. Within the MHC‐II signaling pathway, the apCAF‐like population showed higher centrality in the sender and influencer dimensions, whereas other CAF subtypes did not show the same pattern (Figure 4A).

Chord diagrams showed inferred communication edges originating from the apCAF‐like population in the MHC‐II, TGF‐β and CXCL signaling pathways, connecting to T lymphocytes, B lymphocytes, myeloid cells, and NK cells (Figure 4B).

The ligand–receptor bubble plot derived from the apCAF‐like population showed that MHC‐II‐related pairs mainly included HLA‐C/F/DRA–CD4, TGF‐β‐related pairs mainly included TGFB1/2/3–TGFBR1/2, and CXCL signaling included combinations such as CXCL12–CXCR4/CXCR2; these inferred signals were directed primarily toward myeloid cells, NK cells, B lymphocytes, and T lymphocytes (Figure 4C).

Expression plots of co‐stimulatory molecules showed that, relative to myeloid cells and B lymphocytes, the apCAF‐like population had lower expression of CD80 and CD86, whereas CD40, ICOSLG, TNFSF4, and TNFSF9 were also generally low (Figure 4D). These data define the position of the apCAF‐like population within the MHC‐II‐associated communication network and suggest that it retains antigen‐presentation‐related transcriptional features but limited expression of co‐stimulatory molecules.

### 3.5 Interferon‐response and antigen‐presentation‐associated transcriptional programs in the apCAF‐like population

**FIGURE 5:**
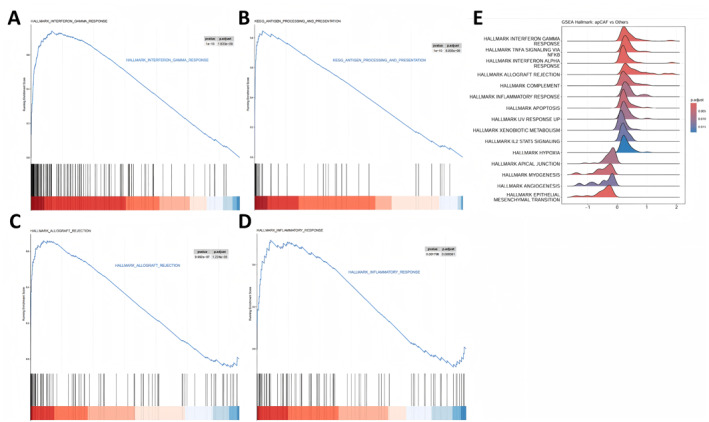
GSEA pathway enrichment landscape of the apCAF‐like population. (A) GSEA enrichment plot of HALLMARK_INTERFERON_GAMMA_RESPONSE in the comparison between the apCAF‐like population and other CAF subtypes. (B) GSEA enrichment plot of KEGG_ANTIGEN_PROCESSING_AND_PRESENTATION. (C) GSEA enrichment plot of HALLMARK_ALLOGRAFT_REJECTION. (D) GSEA enrichment plot of HALLMARK_INFLAMMATORY_RESPONSE. (E) Ridgeplot showing significantly enriched Hallmark gene sets in the apCAF‐like population; the *x* axis indicates fold change and color indicates adjusted *p* value. apCAF, antigen‐presenting cancer‐associated fibroblast; GSEA, gene set enrichment analysis.

GSEA was used to assess pathway enrichment patterns in the apCAF‐like population relative to other CAF subtypes. HALLMARK_INTERFERON_GAMMA_RESPONSE showed positive enrichment in the apCAF‐like population (*p* < 0.0001) (Figure 5A). KEGG_ANTIGEN_PROCESSING_AND_PRESENTATION was also positively enriched (*p* < 0.0001) (Figure 5B).

Additional immune‐related gene sets were likewise represented in the enrichment profile of the apCAF‐like population, including HALLMARK_ALLOGRAFT_REJECTION and HALLMARK_INFLAMMATORY_RESPONSE (all *p* < 0.0001) (Figure 5C,D). These results define the transcriptional features of the apCAF‐like population as being associated with interferon response, antigen processing and presentation, and inflammatory programs.

The ridgeplot of Hallmark gene sets further showed enrichment of pathways, including INTERFERON_GAMMA_RESPONSE, programs related to ANTIGEN_PROCESSING_AND_PRESENTATION, and pathways, such as TNFA_SIGNALING_VIA_NFKB, INTERFERON_ALPHA_RESPONSE, and INFLAMMATORY_RESPONSE (Figure 5E). These data indicate that the apCAF‐like population exhibits a transcriptional state enriched for immune‐related pathways.

### 3.6 Inverse association of KPNB1 expression with immune infiltration and immune score

**FIGURE 6:**
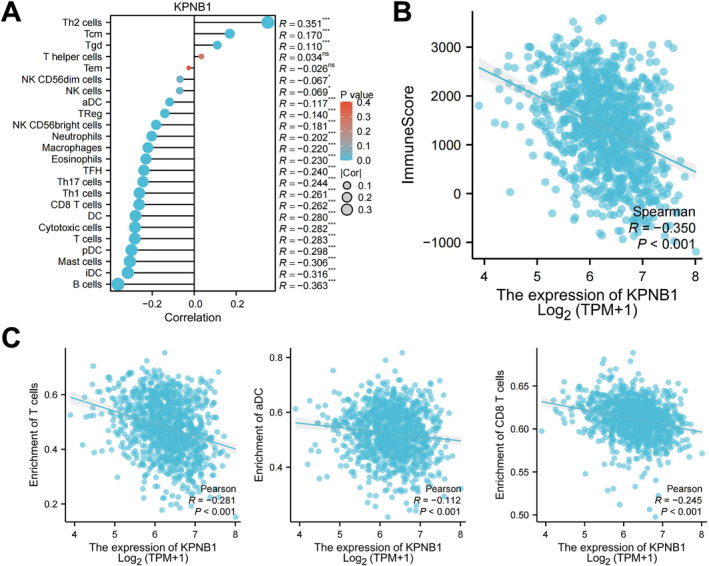
Correlation of KPNB1 expression with immune infiltration and immune score. (A) Lollipop plot showing the correlations between KPNB1 expression and the infiltration scores of immune cell subsets; dot color indicates *p* value and dot size indicates the absolute correlation coefficient. (B) Scatter plot showing the correlation between KPNB1 expression and ImmuneScore; each dot represents one sample, the solid line indicates the fitted curve and the shaded area indicates the 95% confidence interval. (C) Scatter plots showing the correlations between KPNB1 expression and the infiltration scores of T cells, T cells, aDCs, and CD8+ T cells; each dot represents one sample, the solid line indicates the fitted curve and the shaded area indicates the 95% confidence interval. aDCs, activated dendritic cells; KPNB1, karyopherin subunit beta 1.

Immune infiltration features at the bulk transcriptomic level were assessed using ssGSEA and ESTIMATE. Correlation analysis showed that KPNB1 expression was negatively associated with the infiltration scores of multiple immune cell subsets, including B cells, immature dendritic cells, mast cells, plasmacytoid dendritic cells, T cells, cytotoxic cells, dendritic cells (DCs), CD8+ T cells, T helper 1 (Th1) cells, and T follicular helper cells, whereas a limited number of subsets, including Th2 cells, central memory T cells (Tcm cells), and gamma delta T cells (Tgd cells), showed positive correlations (Figure 6A).

The scatter plot of KPNB1 expression versus ImmuneScore showed a negative correlation between the two variables (*p* < 0.001) (Figure 6B). This trend indicates that higher KPNB1 expression corresponds to a lower overall immune score.

Correlation analyses of representative immune cell populations further showed that KPNB1 expression was negatively associated with the infiltration scores of T cells, activated dendritic cell and CD8^+^ T cells (all *p* < 0.001) (Figure 6C). These data define an association between higher KPNB1 expression and lower immune infiltration.

### 3.7 Spatial distribution of KPNB1‐associated spots and their association with immune markers

**FIGURE 7:**
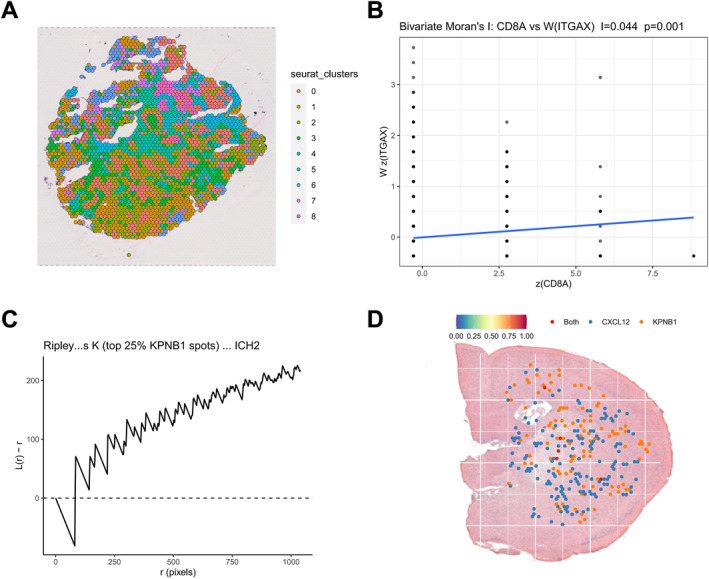
Spatial clustering of KPNB1‐high apCAF‐like signals and their association with immune cells. (A) Spatial clustering plot of the E‐MTAB‐13530 dataset showing the distribution of nine spatial subclusters (Cluster 0–8) across the tissue section. (B) Bivariate Moran's I scatter plot of CD8A and ITGAX; the *x* axis indicates z(CD8A), the *y* axis indicates Wz(ITGAX), and the blue line indicates the linear fit. (C) Ripley's K analysis of KPNB1‐high spots; the *y* axis indicates *L*(*r*) − *r*, the *x* axis indicates spatial radius *r* (pixels), and the dashed line indicates the 0 baseline. (D) Spatial dot plot on the HE background showing the distribution of CXCL12‐high (blue) and KPNB1‐high (orange) spots; red indicates overlapping regions (Both). apCAF, antigen‐presenting cancer‐associated fibroblast; CXCL, C‐X‐C motif chemokine ligand; HE, hematoxylin and eosin; KPNB1, karyopherin subunit beta 1.

Spatial transcriptomic data from E‐MTAB‐13530 were analyzed using Seurat and SPATA2 for spatial clustering and tissue mapping. Unsupervised clustering identified nine spatial subclusters (Cluster 0–8), showing distinct regional distribution patterns across the tissue section (Figure 7A).

Bivariate Moran's I analysis showed a weak positive spatial correlation between CD8A and ITGAX (*p* = 0.001) (Figure 7B). This pattern indicates a degree of spatial proximity between T cell‐related and DC‐related markers.

Ripley's K analysis of KPNB1‐high spots showed that the *L*(*r*) − *r* curve remained above 0 across most spatial scales (Figure 7C). This distribution pattern is consistent with spatial clustering.

Overlay of CXCL12‐high and KPNB1‐high spots on the hematoxylin and eosin background showed partial overlap between the two signals in selected regions (Figure 7D). These data define the spatial clustering pattern of KPNB1‐associated spots and suggest spatial association with immune‐related markers and CXCL12 signaling.

### 3.8 Regional enrichment of KPNB1‐associated spatial modules and antigen‐presentation signals

**FIGURE 8:**
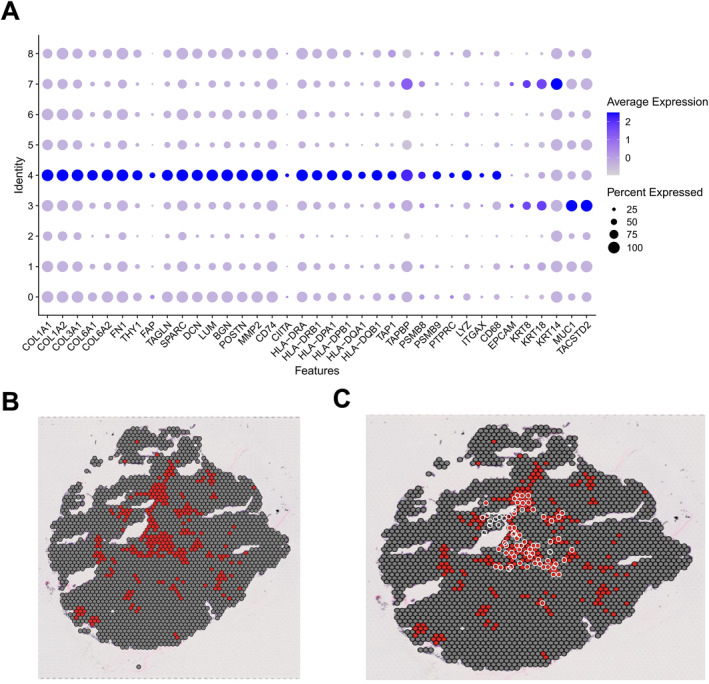
Spatial distribution of KPNB1‐associated modules and their relation to antigen‐presentation signals. (A) Dot plot showing the expression features of representative genes across spatial subclusters; dot color indicates average expression and dot size indicates the proportion of spots expressing each gene. (B) Spatial spot map across the tissue section showing the distribution of the KPNB1‐associated signal; red indicates signal‐positive regions and gray indicates other spots. (C) Spatial spot map across the tissue section showing the distribution of NRF2‐related and antigen‐presentation‐related signals; red indicates signal‐positive regions and gray indicates other spots. KPNB1, karyopherin subunit beta 1; NRF2, nuclear factor erythroid 2‐related factor 2.

Spatial transcriptomic data were used to assess gene‐expression features across spatial subclusters and their tissue localization. The dot plot showed that selected spatial subclusters were enriched for KPNB1, CAF‐related markers, and MHC‐II‐related genes, including HLA‐DRA and HLA‐DRB1 (Figure 8A).

Spatial mapping further showed that the KPNB1‐associated signal was concentrated in specific regions of the tissue section rather than being uniformly distributed across all spots (Figure 8B). This pattern indicates regional enrichment of the KPNB1‐associated module.

Overlay of NRF2‐related and antigen‐presentation‐related signals showed partial spatial overlap between the two in selected regions (Figure 8C). These data define the tissue distribution of the KPNB1‐associated spatial module and suggest local spatial association with antigen‐presentation‐related signals.

### 3.9 NRF2 activity, KPNB1 expression and MHC‐II‐related molecules in CAFs

**FIGURE 9:**
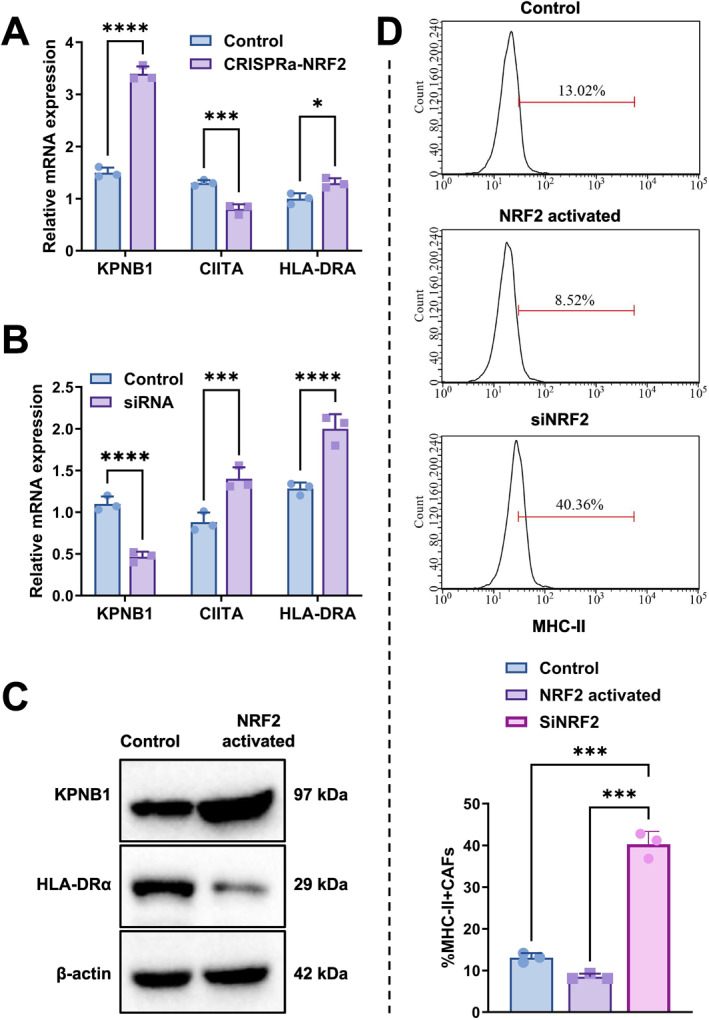
Expression changes of KPNB1 and MHC‐II‐related molecules in CAFs following NRF2 perturbation. (A) Relative mRNA expression of KPNB1, CIITA and HLA‐DRA in primary CAFs following CRISPRa‐mediated NRF2 activation. (B) Relative mRNA expression of KPNB1, CIITA and HLA‐DRA in primary CAFs following siNRF2 treatment. (C) Western blot showing KPNB1, HLA‐DRα and β‐actin protein levels in control and NRF2‐activated CAFs. (D) Flow‐cytometry histograms and quantification showing the proportion of MHC‐II‐positive CAFs in control, NRF2‐activated and siNRF2‐treated groups. Statistical significance is indicated in the figure as follows: *p < 0.05, ***p < 0.001, ****p < 0.0001. CAFs, cancer‐associated fibroblasts; CIITA, class II MHC transactivator; KPNB1, karyopherin subunit beta 1; MHC‐II, major histocompatibility complex class II; NRF2, nuclear factor erythroid 2‐related factor 2.

To validate the regulatory role of NRF2 on the antigen presentation function of CAFs, NRF2 functional intervention experiments were conducted in primary CAFs derived from NSCLC patients. NRF2 activity was perturbed in primary CAFs using CRISPRa and siRNA, followed by assessment of KPNB1 and MHC‐II‐related molecules. CRISPRa‐mediated activation of NRF2 increased KPNB1 mRNA, whereas CIITA and HLA‐DRA mRNA were reduced (all *p* < 0.05) (Figure 9A).

siRNA‐mediated NRF2 knockdown reduced KPNB1 mRNA, whereas CIITA and HLA‐DRA mRNA increased (all *p* < 0.001) (Figure 9B). This pattern was opposite to that observed under NRF2 activation.

At the protein level, NRF2 activation increased KPNB1 and reduced HLA‐DRα (Figure 9C). Flow‐cytometric analysis further showed that NRF2 activation reduced the proportion of MHC‐II‐positive CAFs, whereas siNRF2 increased this fraction (all *p* < 0.001) (Figure 9D). These data define a correspondence between NRF2 activity, increased KPNB1, and reduced MHC‐II‐related molecules in CAFs.

### 3.10 T‐cell activation and cytokine release associated with KPNB1‐low CAFs

**FIGURE 10:**
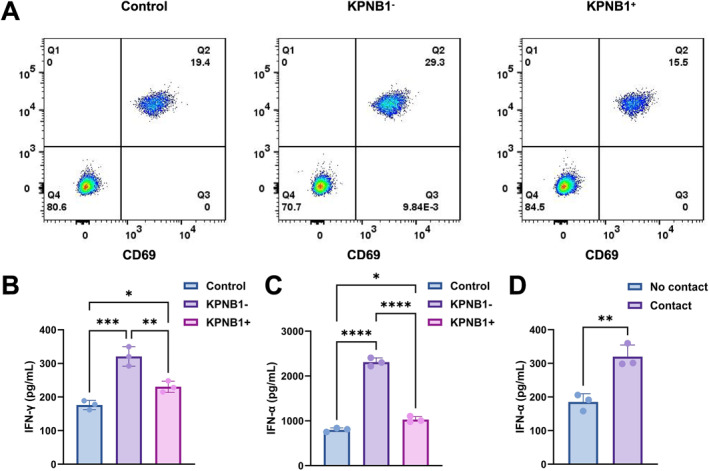
KPNB1‐low CAFs enhance CD8^+^ T‐cell activation and cytokine release. (A) Flow‐cytometry plots of CD8^+^ T cells co‐cultured with control, KPNB1‐low CAF and KPNB1‐high CAF groups, showing the CD69‐ and CD25‐double‐positive population. (B) IFN‐γ levels in co‐culture supernatants from the control, KPNB1‐low CAF and KPNB1‐high CAF groups. (C) A second cytokine readout in the control, KPNB1‐low CAF and KPNB1‐high CAF groups. (D) Comparison of IFN‐α levels between no‐contact and contact conditions in the Transwell assay. Statistical significance is indicated in the figure as follows: *p < 0.05, **p < 0.01, ***p < 0.001, ****p < 0.0001. CAFs, cancer‐associated fibroblasts; IFN‐γ, interferon‐γ; KPNB1, karyopherin subunit beta 1.

Primary NSCLC‐derived CAFs were stratified by KPNB1 expression and co‐cultured with CD8^+^ T cells, followed by assessment of T‐cell activation and cytokine release. Flow cytometry showed that the proportion of CD69^+^CD25^+^ T cells was higher in the KPNB1‐low CAF condition than in the control and KPNB1‐high CAF conditions; the corresponding gated fractions were 29.3%, 19.4% and 15.5%, respectively (Figure 10A).

Cytokine measurements showed that IFN‐γ levels were higher in the KPNB1‐low CAF group than in the control group (*p* < 0.001) (Figure 10B) and the KPNB1‐high CAF group (*p* < 0.01) (Figure 10B), whereas the KPNB1‐high CAF group was lower than the control group (*p* < 0.05) (Figure 10B).

A second cytokine readout showed the same pattern, with the KPNB1‐low CAF group higher than both the control and KPNB1‐high CAF groups (all *p* < 0.0001) (Figure 10C), whereas the KPNB1‐high CAF group also differed from the control group (*p* < 0.05) (Figure 10C).

The Transwell assay showed higher IFN‐α levels under contact conditions than under no‐contact conditions (*p* < 0.01) (Figure 10D). These data define an association between KPNB1‐low CAFs and enhanced T‐cell activation together with increased cytokine release, and suggest a contact‐dependent component of this effect.

### 3.11 Transcriptomic alterations and antigen‐presentation‐associated pathway enrichment following KPNB1 knockdown

**FIGURE 11:**
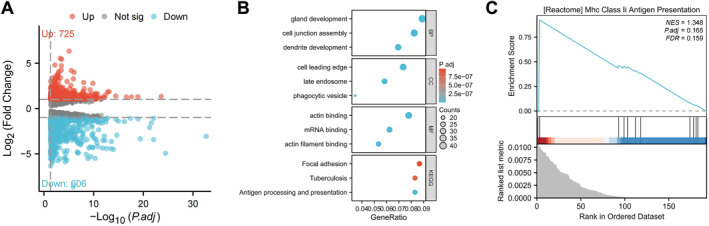
Transcriptomic alterations and antigen‐presentation‐associated pathway enrichment following KPNB1 knockdown. (A) Volcano plot showing DEGs. Red dots indicate upregulated genes (*n* = 725), blue dots indicate downregulated genes (*n* = 606), and gray dots represent genes without significant changes. (B) GO and KEGG enrichment bubble plots highlight significant enrichment of pathways related to MHC‐II complex assembly and antigen presentation. (C) GSEA results demonstrate a positive enrichment trend for the MHC class II antigen presentation pathway among DEGs (NES = 1.348, adjusted *p* = 0.165, FDR = 0.159). DEGs, differentially expressed genes; FDR, false discovery rate; GO, gene ontology; GSEA, gene set enrichment analysis; KEGG, Kyoto encyclopedia of genes and genomes; KPNB1, karyopherin subunit beta 1; MHC‐II, major histocompatibility complex class II.

Transcriptomic changes following KPNB1 knockdown in primary CAFs were assessed by bulk RNA‐seq. Differential expression analysis identified 725 upregulated genes and 606 downregulated genes (Figure 11A).

GO and KEGG enrichment analyses of the DEGs showed that the upregulated genes were mainly enriched for antigen processing and presentation and related functional terms (Figure 11B). These results show that KPNB1 knockdown is accompanied by enrichment of antigen‐presentation‐associated transcriptional programs.

GSEA further showed positive enrichment of the [Reactome] Mhc Class II Antigen Presentation gene set under KPNB1 knockdown, with an normalized enrichment score of 1.348 and an FDR of 0.159 (Figure 11C). This result suggests that KPNB1 knockdown is associated with increased MHC class II antigen‐presentation‐related transcriptional signals.

### 3.12 NRF2–KPNB1 axis inhibition and anti‐PD‐1 treatment response

**FIGURE 12:**
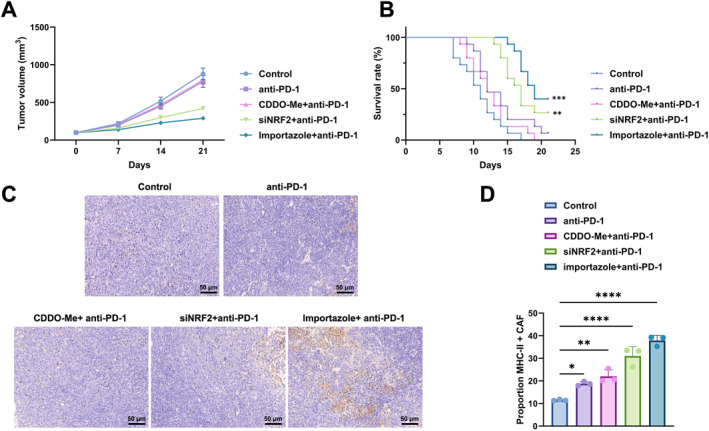
In vivo assessment of NRF2–KPNB1 axis perturbation in combination with anti‐PD‐1 treatment. (A) Tumor growth curves in the orthotopic Lewis lung cancer model across the indicated treatment groups, including control, anti‐PD‐1, CDDO‐Me + anti‐PD‐1, siNRF2 + anti‐PD‐1 and Importazole + anti‐PD‐1. (B) Kaplan–Meier survival curves across the indicated treatment groups. (C) IHC images of tumor tissues from the indicated treatment groups. (D) Quantification of the proportion of MHC‐II^+^ CAFs across the indicated treatment groups. Statistical significance is indicated in the figure as follows: *p < 0.05, **p < 0.01, ***p < 0.001, ****p < 0.0001. anti‐PD‐1, anti‐programmed cell death protein 1; CAFs, cancer‐associated fibroblast; IHC, immunohistochemistry; KPNB1, karyopherin subunit beta 1; MHC‐II, major histocompatibility complex class II; NRF2, nuclear factor erythroid 2‐related factor 2.

An orthotopic Lewis lung cancer model was used to assess the combined effects of NRF2–KPNB1 axis perturbation and anti‐PD‐1 treatment. Tumor growth curves showed limited suppression with anti‐PD‐1 monotherapy, whereas the Importazole + anti‐PD‐1 and siNRF2 + anti‐PD‐1 groups showed slower tumor growth; in contrast, the CDDO‐Me + anti‐PD‐1 group remained close to the control group (Figure 12A).

Kaplan–Meier analysis showed a rightward shift of the survival curves in the Importazole + anti‐PD‐1 and siNRF2 + anti‐PD‐1 groups, whereas the CDDO‐Me + anti‐PD‐1 group did not show the same pattern (Figure 12B). This result was directionally consistent with the tumor growth curves.

IHC images showed differences in staining patterns across treatment groups, with stronger positive staining visible in the Importazole + anti‐PD‐1 and siNRF2 + anti‐PD‐1 groups (Figure 12C). Quantification of the proportion of MHC‐II^+^ CAFs further showed that the Importazole + anti‐PD‐1 and siNRF2 + anti‐PD‐1 groups were higher than the control and anti‐PD‐1 monotherapy groups, whereas the CDDO‐Me + anti‐PD‐1 group was intermediate (comparisons as indicated by the statistical annotations in the figure) (Figure 12D). These data show that the inhibition of the NRF2–KPNB1 axis is associated with enhanced response to anti‐PD‐1 therapy.

## 4 DISCUSSION

By integrating single‐cell transcriptomics, spatial transcriptomics, the TCGA cohort, primary CAF functional assays, and mouse models, the present study identified and characterized an apCAF‐like state with MHC‐II‐associated transcriptional features in NSCLC. This state showed increased expression of antigen‐presentation‐related molecules but limited expression of co‐stimulatory molecules and was associated with the NRF2‐KPNB1 axis, lower immune infiltration, and altered response to anti‐PD‐1 treatment. Recent studies have suggested that CAF heterogeneity and spatial distribution are important determinants of the lung cancer microenvironment and clinical outcome.^44^, ^45^ The current findings further indicate that CAF‐associated immune phenotypes in NSCLC do not represent a uniform suppressive background but may include discrete states linked to local immune tolerance.

The present results were broadly consistent with previous observations that MHC‐II‐associated CAF states can be identified across multiple tumor types and constitute a relatively distinct transcriptional category. A recent review noted that these cells are commonly characterized by expression of CD74 and other MHC‐II‐related molecules, whereas their immunological functions appear to be highly context dependent.^46^ The apCAF‐like state identified here showed the same transcriptional features and occupied a terminal segment of the CAF state‐transition trajectory. These findings supported the view that the apCAF‐like state represents a distinguishable branch within the CAF heterogeneity spectrum, rather than diffuse background expression of individual MHC‐II‐related genes.

Previous studies showed that, in some gastric cancer and pan‐cancer settings, apCAF abundance was associated with immunotherapy sensitivity and proximity to lymphoid structures.^47^ In contrast, the apCAF‐like state identified here was more consistent with an MHC‐II‐associated but co‐stimulation‐limited phenotype, rather than a population directly equivalent to functional antigen‐presenting cells. Tumor type, spatial niche, local inflammatory context, and the tissue relationship between CAFs and immune cells may all influence the immunological output of this state. Expression of MHC‐II molecules alone therefore appeared insufficient to define functional directionality, and local tissue architecture together with parallel signaling cues likely remained important.

Recent studies have associated NRF2 activation with an immunosuppressive lung cancer microenvironment and reduced immune‐cell infiltration.^48^, ^49^ KPNB1, as a nuclear transport protein, participates in the nucleocytoplasmic trafficking of multiple transcriptional regulators, and its aberrant activation has been associated with tumor progression and immune evasion.^31^ In CAFs, NRF2 activation corresponded to increased KPNB1 expression together with reduced CIITA, HLA‐DRA, and the proportion of MHC‐II‐positive cells, whereas NRF2 inhibition showed the opposite pattern. Bulk RNA‐seq following KPNB1 knockdown further showed enrichment of antigen‐presentation‐related pathways. These results supported the possibility that, in CAFs, the NRF2‐KPNB1 axis was more closely linked to the restriction of MHC‐II‐related programs and the maintenance of a tolerogenic phenotype than to effective antigen‐presentation activation.

These observations were also consistent with current understanding of CAF immune biology. Recent work has shown that CAF‐mediated immune suppression is rarely driven by a single pathway, but instead reflects the combined effects of TGF‐β signaling, chemokine networks, extracellular matrix remodeling and interactions with myeloid cells.^50^, ^51^ In the present study, CellChat analysis suggested that the apCAF‐like state participated in MHC‐II‐, TGF‐β‐ and CXCL‐related communication programs while lacking substantial expression of co‐stimulatory molecules. This combination was more compatible with an incomplete antigen‐presentation‐related state than with a classical immune‐activating antigen‐presenting cell. The same framework may also account for the apparent difference between bulk‐level and single‐cell or functional observations: in the TCGA cohort, high KPNB1 expression was associated with lower ImmuneScore and poorer survival outcome, whereas within CAF‐intrinsic analyses, low KPNB1 expression corresponded to higher MHC‐II‐related molecule expression and stronger T‐cell functional readouts. These findings likely reflected the same immune‐phenotypic shift viewed at different analytical scales.

From a translational perspective, the present study supported a shift in CAF‐targeting strategies from wholesale depletion toward functional reprogramming. Recent reviews of CAF biology in NSCLC have emphasized that CAF‐associated immunosuppressive niches are among the major nontumor determinants limiting the efficacy of immune checkpoint blockade.^45^ In the present study, Importazole or siNRF2 in combination with anti‐PD‐1 was associated with slower tumor growth, prolonged survival and a higher proportion of MHC‐II‐positive CAFs, whereas CDDO‐Me did not show the same directional effect. These results suggested that the NRF2‐KPNB1 axis may represent a targetable CAF‐associated node and provided a preclinical rationale for enhancing PD‐1 blockade through CAF phenotypic remodeling.

Several limitations should be noted. First, although single‐cell analysis provided a relatively clear definition of the apCAF‐like state in the core NSCLC dataset, publicly available scRNA‐seq evidence still lacked systematic cross‐cohort validation under a unified analytical framework. Second, spatial transcriptomic analysis was performed at spot resolution rather than single‐cell resolution, such that spatial overlap between KPNB1 and MHC‐II‐related signals could not be interpreted as intracellular co‐expression. Third, although primary CAF perturbation assays and transcriptomic analysis after KPNB1 knockdown established strong molecular and functional associations, direct evidence that KPNB1 regulated MHC‐II programs in CAFs through specific nuclear transport substrates was not obtained. Fourth, in vivo drug and siRNA interventions may also have affected cell populations beyond CAFs; the current animal data were therefore more appropriately interpreted as reflecting axis‐level effects rather than CAF‐specific causality. Fifth, multiplex immunofluorescence and higher‐dimensional spatial validation were not included, and the spatial heterogeneity and functional states of CAFs will require further evaluation using higher‐resolution spatial approaches.^44^, ^45^

Future work may proceed in three directions. First, single‐cell spatial approaches, multiplex immunofluorescence and protein‐level validation may refine the cellular composition and tissue organization of the apCAF‐like niche. Second, CAF‐specific genetic perturbation and tracing of nuclear transport substrates may help define how the NRF2‐KPNB1 axis regulates CIITA, MHC‐II‐related programs and immune output within CAFs. Third, larger clinical cohorts may be used to assess the relationship between KPNB1‐associated CAF phenotypes and anti‐PD‐1 response, and to explore combination strategies involving additional immune checkpoints or stromal remodeling approaches. Recent studies have repeatedly suggested that CAF targeting is more likely to achieve clinical value through state reprogramming than through nonselective depletion.^45^, ^51^, ^52^ The present findings supported this direction and provided a basis for further mechanistic and translational investigation.

## AUTHOR CONTRIBUTIONS

Fei Zheng conceived and designed the study; Ruoying Deng and Ran Hou performed the experiments and analyzed the data; Lei Hong and Yanzhi Cui contributed to methodology, validation, and investigation; Yibing Liu provided critical resources and supervised the project. The manuscript was written and reviewed by Fei Zheng, Ruoying Deng, and Ran Hou with input from all authors. All authors have read and agreed to the published version of the manuscript.

## CONFLICT OF INTEREST STATEMENT

The authors declare no conflicts of interest.

## ETHICS STATEMENT

All animal experiments were approved by the Animal Ethics Committee of the Fourth Hospital of Hebei Medical University.

## Supporting information

Figure S1

## Data Availability

All data generated or analyzed during this study are included in this article and/or its supporting information. Further enquiries can be directed to the corresponding author.
