# Supplementary material for: Multi‐omics analysis identifies a major histocompatibility complex class II‐associated antigen‐presenting cancer‐associated fibroblast‐like state linked to the nuclear factor erythroid 2‐related factor 2‐karyopherin subunit beta 1 axis in nonsmall cell lung cancer
Source: J Cell Commun Signal. 2026 Jun 4;20(2):e70074. doi: 10.1002/ccs3.70074 (PMC13238553; doi:10.1002/ccs3.70074)
Supplement: Supplementary file 1 — Figure S1 [file CCS3-20-e70074-s001.docx]

**
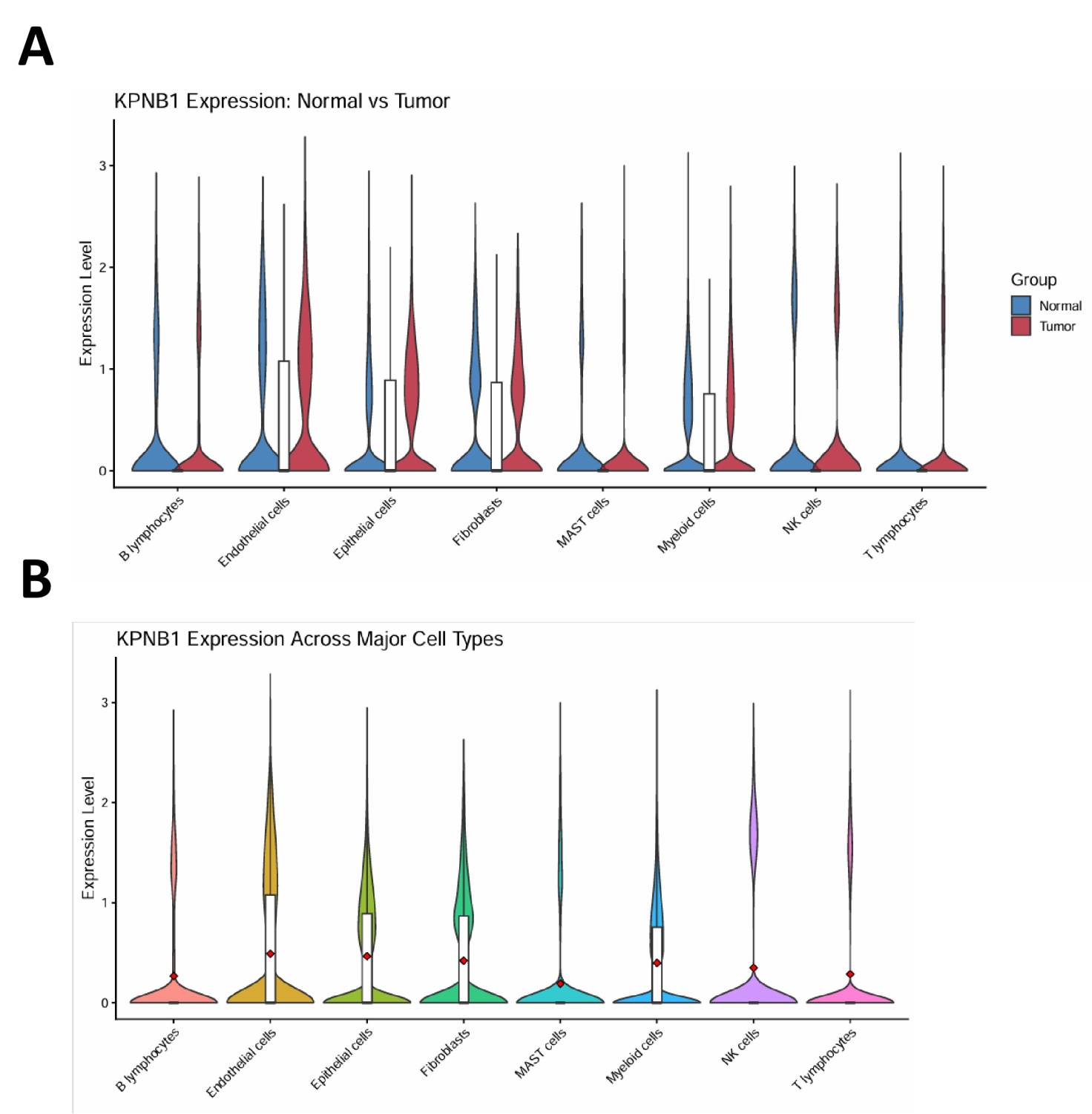
**

**Figure S1. Distribution of KPNB1 expression across major cell types in NSCLC.**

Note: (A) Split violin plots showing KPNB1 expression across major cell types in normal and tumour tissues; blue indicates normal tissue and red indicates tumour tissue. (B) Violin plots showing KPNB1 expression across major cell types in the tumour microenvironment; red dots indicate mean expression levels.
